# *Lactobacillus reuteri* Promotes Intestinal Development and Regulates Mucosal Immune Function in Newborn Piglets

**DOI:** 10.3389/fvets.2020.00042

**Published:** 2020-02-07

**Authors:** Minjuan Wang, Haiqin Wu, Linhao Lu, Lan Jiang, Qinghua Yu

**Affiliations:** MOE Joint International Research Laboratory of Animal Health and Food Safety, College of Veterinary Medicine, Nanjing Agricultural University, Nanjing, China

**Keywords:** *Lactobacillus*, piglets, intestinal development, mucosal immunity, proliferation

## Abstract

Intestinal microbiota is necessary for the guarantee of intestinal mucosal barrier. However, the detailed effect of probiotics on porcine intestinal development, especially in the early life, is still unclear. In this study, we treated 3-day-old newborn piglets with *Lactobacillus reuteri* (*L. reuteri*) D8 and observed its beneficial effect on piglets. The body weights, villus height, and crypt depth of jejunum were all significantly increased after *L. reuteri* treatment in piglets. *L. reuteri* also significantly increased the proliferation index of PCNA^+^ cells in the crypt, as well as c-Myc and Tcf4 expressions. Furthermore, *L. reuteri* also enhanced intestinal mucosal barrier with the increase of goblet cells and antimicrobial peptides (AMPs) expressions of Muc2, Lyz1, and pBD1. The well development of Peyer's patches and increased number of CD3^+^ T cells, combined with increased expression of IL-4 and IFN-γ, also demonstrated the immune stimulation effect of *L. reuteri* D8. This study demonstrated that *L. reuteri* promotes the development of intestine mucosal system and maintains intestinal mucosal barrier in newborn piglets.

## Introduction

Piglets diarrhea with intestinal inflammation and epithelial damage is a common problem and thus contributes to morbidity and mortality among piglets, particularly in intensive farming systems (1). The development of the gastrointestinal tract in newborn piglets is a very sophisticated process, which starts during prenatal life and continues after birth. The intestinal development of newborn piglets is not perfect and vulnerable to the invasion of various pathogens (2). Numerous studies have shown high morbidity of diarrhea and intestinal inflammation in piglets, resulting in the death of piglets and directly causing great economic losses (3, 4). The addition of antibiotics to livestock and poultry feed can significantly improve the feed conversion rate, the growth rate, and reduce the mortality rate (5–7). However, this phenomenon will cause bacterial drug resistance, seriously destroy the immune mechanism of the body, and weaken the ability of anti-virus and anti-infection. Moreover, antibiotics accumulate in the body, endangers human health, and ecological environment (8, 9).

The intestine is not only a major digestive and absorptive organ for nutrients, but also an effective barrier against various infectious agents. The intestinal mucosal barrier mainly contains mucus layers, epithelial cells, and mucosal immune system (10). The intestinal epithelium bind tightly together to form a dense protective wall, separating the intestinal contents from the intestinal tissues, and maintaining the health of intestinal tract (11). Once the intestinal mucosal barrier is destroyed or the balance of intestinal microflora is broken, it will lead to an increase of intestinal epithelial permeability, the destruction of the intestinal structure, and the displacement of pathogens that turn into other organs such as the liver, thus causing a systemic pathological response (12, 13).

Probiotics are live microbial food supplements or components of bacteria which have been shown beneficial effects on health (14). At present, probiotics are becoming substitutes for antibiotics, such as *Lactobacillus, Bifidobacterium*, and *Bacillus*. In previous study, it has been found that *Lactobacillus* can reduce the alkaline environment in intestinal tract, increase mucus secretion and strengthen the tight connection, so as to maintain the homeostasis of the internal environment (15–17). However, the mechanism of *Lactobacillus* on intestinal development and mucosal barrier in newborn piglets is not clear. *Lactobacillus reuteri* (*L. reuteri*) is one of the dominant species in the GIT of pigs (18). In this study, we hypothesis whether *L. reuteri* can affect intestinal stem cells and mucosal immune responses of piglets, which may provide a powerful basis for the application of *L. reuteri* in pig raising.

## Materials and Methods

### Animals and Bacteria Strains

Twelve 3-day-old piglets were purchased from Meishan Pig original breeding Farm in Jiangsu Province. The piglets were fed with sows in the laboratory animal room of Nanjing Agricultural University. The initial weight and health status of piglets were equal and piglets were male. *L. reuteri* D8 was isolated from the duodenum of pigs in our laboratory and further confirmed as *L. reuteri* strain through 16s RNA sequencing (GenBank: MF850249), which are tetracycline resistance. *L. reuteri* D8 were grown in MRS ager medium at 37°C (19). Pigs were raised in the laboratory animal room of Nanjing Agricultural University and divided into control group (6 piglets) and *L. reuteri* treatment group (6 piglets). The piglets were treated with 2 mL aseptic PBS or *L. reuteri* D8 (10^9^ CFU) suspended in 2 mL PBS by gavage for 5 days and then sacrificed (20–22). The body weights of the pigs were recorded daily. Harvested jejunum and ileum tissues were fixed with 4% paraformaldehyde. Animal procedure was carried out in accordance with the Nanjing Agricultural University of Medicine Animal Studies Committee, which approved the protocols.

### Histological Analysis

Tissue samples from the small intestine were fixed in 4% paraformaldehyde for 48 h at room temperature. After fixation, the samples were sectioned to fit glass slides and then dehydrated in a graded alcohol series. Then the tissue block is transparent in xylene, soaked in paraffin for 2 h and embedded in paraffin. The sections were serially sliced into 5-μm-thick sections, and mounted on slides. The sections were dried horizontally on a warming tray overnight at 37°C, and stained with hematoxylin-eosin (HE) for examination by light microscopy. The villus height and crypt depth of jejunum were measured (single-blind) by an observer using computer-assisted morphometry. The area of Peyer's patches (PPs) in ileum was also measured.

### Immunohistochemistry

Paraffin sections were dewaxed in xylene and rehydrated in decreasing concentrations of ethanol. The sections were permeabilized with 0.5% Triton X-100 for 15 min, followed by washing three times with HBSS, then dipped in H_2_O_2_ (3%) to remove catalase. For PCNA staining, sections were incubated with anti-porcine PCNA antibody (1:100 Abcam) overnight at 4°C in a humidified box. For CD3 positive T cells staining, sections were incubated with anti-porcine CD3 (1:200 Abcam) overnight at 4°C. PBS was used instead of antibody for the control. Sections were then incubated goat against Rabbit (1:200 Boster) for 1 h at 37°C and washed. DAB color development for 5-10 min. In this study, nearly 50 sections were observed per group. PCNA positive cells and CD3 positive T cells of per crypt in jejunum were measured.

### Periodic Acid-Schiff (PAS) Stain

Jejunum sections were dewaxed in xylene and rehydrated in decreasing concentrations of ethanol. Deparaffinized and rehydrated sections were treated with periodic acid for 5 min at RT. Slides were washed in distilled water then stained with Schiff's reagent for 15 min at RT, followed by a 10 min wash in running tap water. The sections were then counterstained with hematoxylin for 2 min and acid differentiation solution for 3 s. The sections were washed in running tap water for 15 min, followed by dehydration (75, 85, 95, 100%, EtOH) and cover-slipped. The number of goblets cells were counted in the jejunum.

### ELISA

The collected blood was centrifuged at 8,000 rpm for 10 min. The supernatants were stored at −20°C until use. Porcine LPS (SBJ-z255, Sbjbio) and IgG (SBJ-z124, Sbjbio) in serum were measured with ELISA kits according to the manufacturer's instructions.

### Real-Time PCR Quantification

RNA was extracted from jejunum tissues, which were cut into 2 mm size and put in a lapping tube fitted with 500 μL RNAios Plus (Takara), respectively. The tubes were grinded for 1 min and centrifuged at 8,000 g for 10 min at 4°C. After centrifugation, the supernatant was transferred to other tubes and 500 μL RNAios Plus (Takara) was added. Next, 200 μL of chloroform was added and mixed by hand during 15 s and placed at room temperature for 10 min. The tubes were centrifuged at 12,000 g for 15 min at 4°C. After centrifugation, the supernatant (400 μL) containing RNA was transferred to other tubes, then the same volume of isopropanol was added. The tubes were mixed by hand for 15 s and incubated overnight at −20°C. The next day, the tubes were centrifuged at 12,000 g for 20 min at 4°C. The supernatant was discarded, 1 mL of 75% ethanol was added twice and centrifuged at 12,000 g for 10 min at 4°C. Finally, 75% ethanol was discarded and the tubes was dried. The RNA precipitate was dissolved in 20 μL of RNase free water. The CDNA was synthesized by RNA, according to the instructions for the use of manufacturer's enzymes (Takara). Gene expression was assessed with a SYBR master mix (Takara). Relative mRNA expression was calculated using the ΔΔCt method. Primer sequences for real-time RT-PCR were as follows in Table 1.

**Table 1:** Primer sequences used for RT-qPCR.

**Target genes**	**Primer sense (5^**′**^-3^**′**^)**	**Primer antisense (5^**′**^-3^**′**^)**
Lyz-1	AACTGCTTTGGGTGTCTTGC	GGTCTATGATCGGTGCGAGT
Muc2	CTCAGCCGGGATCCAATCTC	GAAAGCCCCGGTGTAACCAT
c-Myc	CTCGGACTCTCTGCTCTCCT	TTGTTCTTCCTCAGAGTCGCT
Lgr5	GCTGGCTGCCGTGGATGC	AGCAGGGCGCAGAGGACAAG
Tcf4	TAATGGAGCAAAGGGTCGTC	CCTGGTTTGGGACAAGAGAA
IL-4,	GCCGGGCCTCGACTGT	TCCGCTCAGGAGGCTCTTC
IFN-γ	TGGTAGCTCTGGGAAACTGAATG	TGGCTTTGCGCTGGATCT
GAPDH	AGGTCGGAGTGAACGGA	TGGGTGGAATCATACTGG

### Statistical Analysis

All data are expressed as means ± SD. The data were analyzed by One-Way ANOVA and Student's *t*-test with SPSS17.0 Software. Significance levels are indicated as ^*^P < 0.05 and ^**^ *P* < 0.01.

## Results

### Effects of *L. reuteri* D8 Treatment on Body Weight and Jejunum Morphology of Piglets

**Figure 1:**
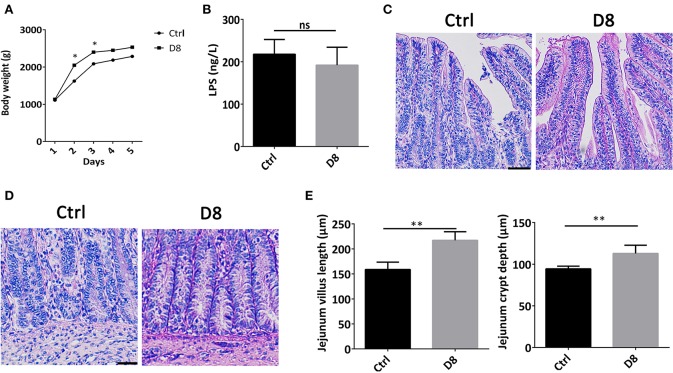
Effects of *L. reuteri* D8 treatment on body weight and jejunum morphology of piglets. **(A)** The body weight of piglets in the two groups were shown. The line chart indicated the body weight of piglets were measured by gram, *n* = 6. **(B)** The level of LPS in serum was detected, *n* = 6. **(C,D)** HE staining of piglet jejunum treated with PBS or *L. reuteri* D8, respectively, *n* = 50 villus or crypts per group. Scale bars, 50 μm. **(E)** Statistical graph of jejunal villus and crypt depth in piglets. Data were expressed as mean ± standard deviation (means ± SD). *P < 0.05, **P < 0.01.

The body weights of piglets in control group and *L. reuteri* D8 treatment group were observed during experimental period. Compared with control group, the body weights of *L. reuteri* D8 treatment showed a significant increase from day 1 to day 3 (Figure 1A). *L. reuteri* D8 didn't affect LPS levels in serum compared to the control group (Figure 1B). The villus length and crypt depth of the jejunum in the *L. reuteri* treatment group were also longer than that in the control group (Figures 1C–E). These results demonstrated that *L. reuteri* D8 treatment increased body weight of piglets and promoted growth of intestinal epithelium to maintain the intestinal mucosal barrier.

### Proliferation Status of the Jejunum After *L. reuteri* D8 Administration

**Figure 2:**
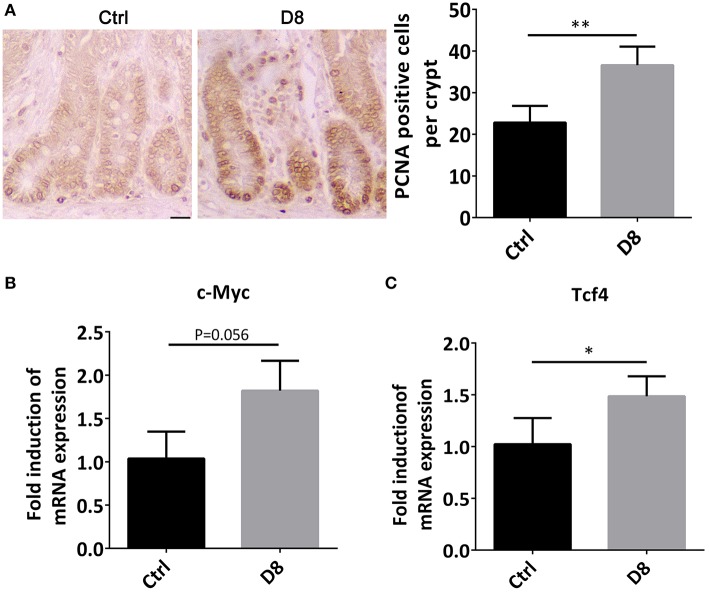
Proliferation status of jejunum after *L. reuteri* D8 administration. **(A)** Immunohistochemistry showed the number of anti-porcine PCNA-positive cells in the jejunal crypt of each group, *n* = 50 crypts per group. Scale bars, 50 μm. **(B,C)** Real-time PCR showed the expression of c-Myc and Tcf4 genes in the jejunum, *n* = 6. Data were expressed as mean ± standard deviation (means ± SD). *P < 0.05, **P < 0.01.

The jejunum was stained by PCNA immune-histochemical staining, and observed under a confocal laser microscope. The proliferative index was significantly higher in *L. reuteri* D8 administration compared with control group (Figure 2A). At the same time, we also found that the mRNA level of c-Myc in jejunum was slightly up-regulated (Figure 2B). After *L. reuteri* D8 administration, Tcf4 mRNA levels had the expected increase (Figure 2C). β-Catenin plays essential roles in the Wnt pathway by interacting with T-cell factor 4 (TCF4) to transcribe oncogenes (23). Researches showed activation of wnt/β-catenin signaling pathway can promote the proliferation of intestinal stem cells (24, 25). The data indicate that *L. reuteri* D8 enhanced intestinal epithelial proliferation by activation of wnt/β-catenin pathway.

### *L. reuteri* D8 Administration Improved Innate Immunity of Piglets

**Figure 3:**
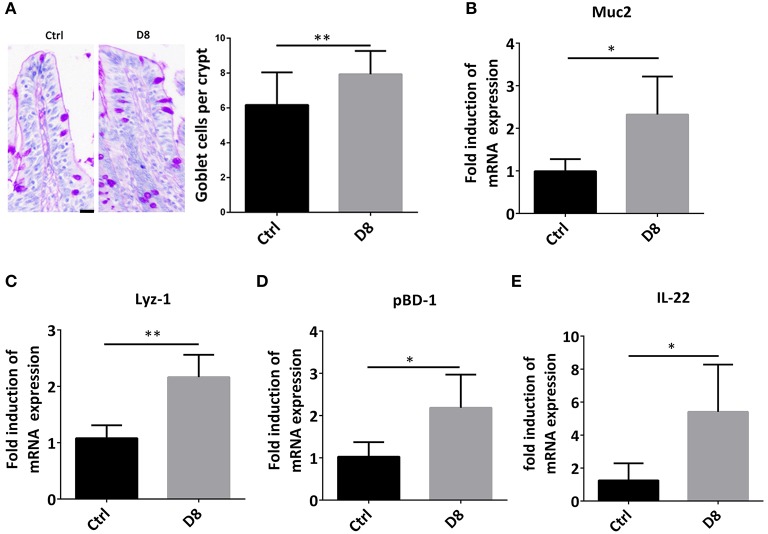
*L. reuteri* D8 administration improved innate immunity of piglets. **(A)** PAS staining of goblet cells in jejunum, *n* = 50 crypts per group. Scale bars, 25 μm. **(B–E)** Real-time PCR was used to determine the relative mRNA expression of muc2, Lyz-1, pBD1, and IL-22 in the jejunum treatment with PBS or *L. reuteri* D8, respectively, *n* = 6. Data were expressed as mean ± standard deviation (means ± SD). *P < 0.05, **P < 0.01.

Intestinal stem cells can differentiate into secretory cells, such as goblet cells and Paneth cells, which can secrete antimicrobial peptides to play an important role in innate immunity against infection (26, 27). In order to verify the effect of *L. reuteri* D8 on immune response in piglets, we detected the number of goblet cells, the expression of Muc2 and Lyz-1. The study demonstrated that *L. reuteri* D8 administration significantly increased the number of goblet cells, the expression of Muc2 and Lyz-1 in jejunum (Figures 3A–C). *L. reuteri* D8 also significantly increased the mRNA expression level of porcine β-defensins 1 (pBD-1) in jejunum (Figure 3D). After *L. reuteri* D8 treatment, the expression of IL-22 mRNA was the quadruple of untreated group (Figure 3E). Based on these data, *L. reuteri* D8 promoted the differentiation of intestinal stem cells and stimulated innate immunity.

### Activation of Mucosal Immunity After *L. reuteri* D8 Administration in Piglets

**Figure 4:**
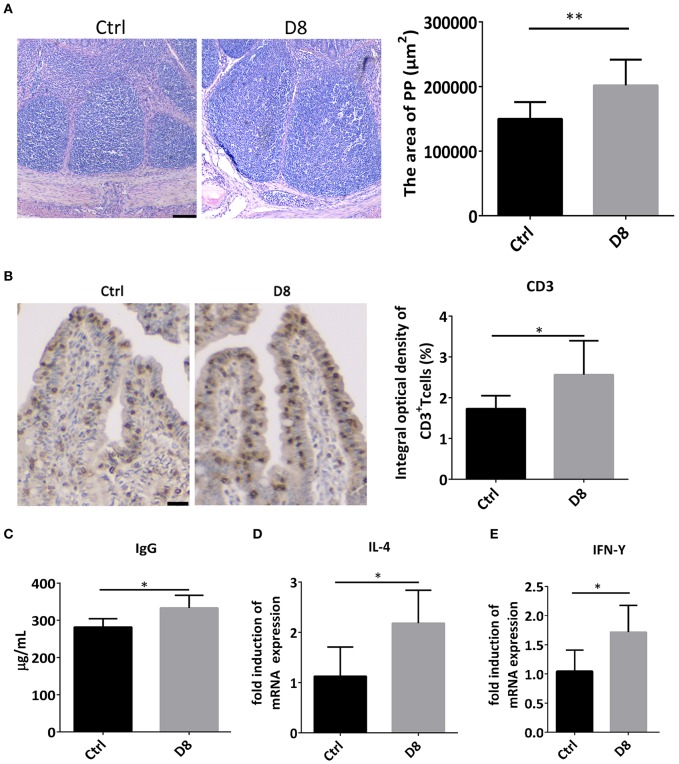
Expression of cytokines after *L. reuteri* D8 administration in piglets. **(A)** HE staining showed the area of the PPs of the piglet's in ileum treat with PBS or *L. reuteri* D8, respectively, *n* = 30 PPs per group. Scale bars, 200 μm. **(B)** Immunohistochemistry of CD3^+^ T cells in jejunum; *n* = 50 crypts per group. Scale bars, 25 μm. **(C)** The level of IgG antibody in serum; *n* = 6. **(D,E)** Real-time PCR detection of the gene expression levels of IL-4 and IFN-γ in jejunum, *n* = 6. Data were expressed as mean ± standard deviation (means ± SD). *P < 0.05, **P < 0.01.

Peyer's patch, also known as intestinal collecting lymph node, is an important part of intestinal mucosal immune system and a group of lymphoid follicles in small intestinal mucosa (28). In this study, the HE staining showed *L. reuteri* D8 treatment markedly increased the area of Peyer's Patches in ileum (Figure 4A). Moreover, immunohistochemistry showed that the number of CD3^+^ T cells observably increased in *L. reuteri* treatment group in jejunum (Figure 4B). Meanwhile, *L. reuteri* D8 treatment also increased the level of IgG antibodies in the serum (Figure 4C). IL-4 induces the differentiation of naïve CD4 T cells into Th2 cells, while IL-4 also drives the immunoglobulin (Ig) class switch to IgG1 and IgE (29). IFN-γ produced by activated Th1 cell, can regulate the immune system, resist viral and bacterial infections and tumorigenesis (30). IL-4 and IFN-γ mRNA levels had a pronounced increase, compared to the control group after *L. reuteri* D8 treatment (Figures 4D,E). These data suggested that *L. reuteri* D8 enhanced intestinal immune response.

## Discussion

Probiotics has already been widely used in pig industry, accompanied with the restriction use of antibiotics. However, the detail mechanism between *Lactobacillus* and intestinal epithelial, especially for the intestine development in early life, is still covered. This study was conducted to investigate the effects of *L. reuteri* D8 in newborn pigs, with respect to growth performance, intestinal morphology, the proliferation of intestinal stem cell and development of innate and mucosal immunity. The formation of villi and crypt in the small intestine significantly enlarged the surface area of the intestinal mucosa, promoted the efficient absorption of nutrient and generated a protected stem cell niche (31–33). In neonate piglets, the integrity of the intestinal mucosal barrier has defectiveness in order to increase the absorption efficiency, especially for antibodies in milk. However, the imperfect intestine is also the infection target of pathogens, such as *Escherichia coli* and porcine epidemic diarrhea virus (34, 35). Stimulating the intestinal development and enhancing the intestinal mucosal ability are necessary for the health raising of pigs. In this study, we found that *L. reuteri* D8 could stimulate the body weight slightly with the supportive of increased villus height and crypt depth in jejunum of piglets. The stimulation effect of *L. reuteri* D8 on intestinal epithelial was further verified with the PCNA staining, as well as c-Myc and Tcf mRNA expressions. The increased villi height will provide more surface area of intestinal lumen to guarantee the absorption of more nutrients, which could also explain the increased body weight by *L. reuteri* D8 in early life.

Intestinal mucosal innate immunity constitutes the first line of defense, fundamental for the recognition and the initiation of an inflammatory response against microorganisms (36). The intestinal epithelial is composed of four cell lineages that come from a common stem cell progenitor: absorptive enterocytes, mucus-producing goblet cells, hormone-producing enteroendocrine cells, and Paneth cells, which produce antimicrobial peptides (37). In this study, we demonstrated that *L. reuteri* D8 successfully enhanced the number of goblet cells and the mRNA expression levels of Muc2 and Lyz-1, which are secreted by goblet cells and Paneth cells, respectively. The stimulatory effect of *Lactobacillus* on Muc-2 expression was consistent with previous study (38).

Besides Muc2 and lysozyme, pBD1, the first member of porcine β defensin, has a strong inhibitory activity against gram-negative bacteria (39). *L. reuteri* D8 also showed stimulatory effect on pBD1, as well as IL-22 expression. IL-22 serves as a communication line between the immune system and the epithelial cells in contact with the outside world (40). Previous study already demonstrated IL-22 secreted from LPLs could accelerate proliferation of intestinal epithelial, thus recovering damaged intestinal mucosa (19). Antimicrobial peptides are effector molecules of innate immunity with direct antimicrobial activity (41). Combined with this increased AMPs induced in early life of piglets, *L. reuteri* D8 showed a strong stimulation effect on enhancing the intestinal mucosal innate immunity. Antimicrobial factors produced by the intestinal mucosa limit the translocation of pathogenic microbes across the intestinal epithelial cell barrier (40).

The gut-associated lymphoid tissue (GALT) consists of both isolated and aggregated lymphoid follicles, while Peyer's Patches are composed by aggregated lymphoid follicles and PPs sampling of the lumen is crucial for protective mucosal immune responses (42). Here, *L. reuteri* D8 increased the area of PPs in ileum of piglets, which indicated the stimulation of immune tissue development and explained the enhanced serum IgG concentration. This phenomenon was further verified with the increased the number of CD3^+^ T cells and expression of IL-4 and IFN-γ, which are necessary for naive T cells differentiation to further increased the intestinal immunity (43, 44).

In summary, this study demonstrated that administration of *L. reuteri* is helpful to stimulate the intestinal development with increased intestinal villi and intestinal crypt, as well as PCNA expression. *L. reuteri* also increased AMPs expression and Peyer's Patches development to enhance the intestinal innate immunity for piglets in early life.

## Data Availability Statement

All datasets generated for this study are included in the article/supplementary material.

## Ethics Statement

The animal study was reviewed and approved by Nanjing Agricultural University of Medicine Animal Studies Committee.

## Author Contributions

MW and HW were responsible for performing the experiments, data analysis, and writing the manuscript. LL and LJ provided help for the animal study. QY were responsible for the conception and design of the study, data collection, drafting the article, and final approval of the version submitted.

### Conflict of Interest

The authors declare that the research was conducted in the absence of any commercial or financial relationships that could be construed as a potential conflict of interest.
